# Delivering Trio Germline Whole Genome Sequencing to Patients Newly Diagnosed With Childhood Cancer: Healthcare Professionals' Perspectives of the PREDICT Study

**DOI:** 10.1002/cam4.70680

**Published:** 2025-02-14

**Authors:** Jacqueline D. Hunter, Kate Hetherington, Claire E. Wakefield, Katherine M. Tucker, Brittany C. McGill, Andrew Grant, Noemi A. Fuentes‐Bolanos, Bhavna Padhye, Margaret Gleeson, Kanika Bhatia, Michelle Peate

**Affiliations:** ^1^ Department of Obstetrics, Gynaecology and Newborn Health, Royal Women's Hospital, Melbourne Medical School, Faculty of Medicine, Dentistry and Health Sciences University of Melbourne Melbourne Victoria Australia; ^2^ School of Clinical Medicine, Randwick Clinical Campus, Discipline of Paediatrics and Child Health UNSW Sydney Sydney New South Wales Australia; ^3^ Behavioural Sciences Unit, Kids Cancer Centre Sydney Children's Hospital Sydney New South Wales Australia; ^4^ Hereditary Cancer Centre, Department of Medical Oncology Prince of Wales Hospital Randwick New South Wales Australia; ^5^ Prince of Wales Clinical School UNSW Sydney Randwick New South Wales Australia; ^6^ Kids Cancer Centre Sydney Children's Hospital Sydney New South Wales Australia; ^7^ School of Nursing and Midwifery, Faculty of Health University of Technology Sydney Sydney New South Wales Australia; ^8^ Children's Cancer Institute, Lowy Cancer Research Centre UNSW Sydney Sydney New South Wales Australia; ^9^ Cancer Centre for Children The Children's Hospital at Westmead Westmead New South Wales Australia; ^10^ Kids Research, Children's Cancer Research Unit The Children's Hospital at Westmead Westmead New South Wales Australia; ^11^ Children's Cancer and Haematology Service John Hunter Children's Hospital Newcastle New South Wales Australia; ^12^ Royal Children's Hospital Melbourne Victoria Australia

**Keywords:** cancer genetics, genome wide sequencing, hereditary cancer, pediatric cancer

## Abstract

**Background:**

Germline genomic sequencing (GS) is increasingly offered to children with cancer. To optimize integration into routine care, assessment of implementation barriers and a better understanding of healthcare professionals' perspectives and experiences are needed.

**Methods:**

Healthcare professionals delivered trio germline GS to newly diagnosed pediatric and adolescent patients with cancer via the PREDICT completed questionnaires with qualitative and quantitative items. Each study site recorded reasons for eligible families' nonenrolment in PREDICT to identify barriers to recruitment. Quantitative data were analyzed via descriptive statistics, whereas qualitative data underwent inductive content analysis, with results integrated for interpretation.

**Results:**

Thirty‐three healthcare professionals participated, including 23 oncology professionals and 10 genetic professionals. Healthcare professionals perceived PREDICT as beneficial to participating and future families, and that perceptions of personal benefit and altruism were drivers of family uptake. Concerns included workforce capacity and potential family distress given the trio design and high‐stress diagnosis setting. Barriers to recruitment related to clinical decision‐making, family factors, and logistics. Although most rated their genetics/genomics knowledge as “good,” regarding germline results, few were “very confident” interpreting (29%), explaining (32%), making treatment recommendations (9.7%), and providing psychosocial support to families (29%). They acknowledged a need for further training in these areas for trainees; yet, fewer were interested in training for themselves.

**Conclusion:**

Successful implementation of routine germline GS will require targeted strategies to address logistical issues and alleviate potential negative psychosocial impacts for families. Recognizing the escalating demand on genetics experts, upskilling of the current workforce and involvement of a broader spectrum of healthcare professionals are warranted.

## 1 Introduction

Recent large‐scale genomic sequencing (GS) studies estimate rates of cancer‐predisposing germline pathogenic or likely pathogenic variants (P/LPV) in patients with childhood cancer between 8% and 18% [1, 2, 3, 4]. As understanding of the genomic landscape of cancer evolves, this number will likely increase. Benefits of identifying cancer predisposition in children with cancer are extensive. A cancer predisposition diagnosis may help families understand the causes of their child's cancer [5], guide treatment, and inform further testing, reproductive decisions, and cancer surveillance for the child and their other at‐risk relatives [6]. To identify cancer predisposition in children, germline GS is increasingly offered alongside somatic GS as part of precision oncology programs [4, 7, 8, 9, 10, 11, 12]. Germline GS as part of precision oncology is predicted to become the standard of care for all newly diagnosed children with cancer, necessitating a workforce adept to sustainably deliver this new technology.

Existing research suggests healthcare professionals and families hold positive attitudes toward germline and somatic GS in the context of precision oncology [13, 14, 15, 16, 17]. However, parents have described difficulties comprehending distinctions between germline and somatic GS when offered together, supporting differentiation of these processes [18]. This becomes particularly significant when considering a trio‐based approach, which involves analyzing germlines of biological parent–child pairs. Trio GS in childhood cancer is favorable to singleton sequencing for its improved diagnostic efficacy and ability to inform inheritance patterns and diagnose clinically relevant conditions like parental mosaicism [19, 20]. However, trio germline GS can introduce complexity for families with consequences beyond cancer care for their child [19]. Additionally, obtaining consent and samples from parent–child pairs may be time‐consuming, costly, and logistically difficult. Although studies exploring potential barriers to implementation or healthcare professional perspectives within trio contexts are limited, initial reports indicate strong interest among families [21].

Despite growing support from healthcare professionals and families toward germline GS in childhood cancer, healthcare professionals delivering this testing face challenges. Genomics has ushered in a new way of working for medical professionals [14], including increased demand on nongenetic professionals to order, understand, and explain complex genomic tests. Current evidence suggests that the oncology workforce is not yet equipped to fulfill these new demands [14, 16, 22, 23]. Previous studies examining pediatric oncologists' experiences of returning GS results to families with a child with cancer found limited clinician confidence and knowledge in interpreting or explaining germline genomic results [24]. Pediatric oncologists have also described challenges in conveying uncertain genomic information in lay language and navigating emotional impacts for families [25].

Genetic professionals are typically embedded in interdisciplinary research teams delivering germline GS to children with cancer. They are well equipped to return and explain germline findings to families and are valued by pediatric oncologists, who have described relying on them to support the return of germline results [25, 26]. While this increased interdisciplinary engagement is valuable, overreliance on genetic professionals to recruit, consent, and return germline GS results may lead to an unsustainable burden on clinical genetic services facing increasing demand [27, 28]. A clear plan for how the workforce will coordinate germline GS as it moves toward integration into standard practice, which may require support from other professionals [29], is needed.

Previous studies have focused on the experiences of clinicians delivering paired germline and somatic GS as part of precision medicine [13, 14, 15, 16, 30]. There is limited research exploring barriers to patient recruitment or healthcare professionals' experiences and perspectives toward standalone germline GS for childhood cancer, or when delivered as trio testing, at diagnosis. Understanding these experiences will allow for the development of appropriate models of care that maximize patient and family engagement, satisfaction, and overall clinical benefit. A better understanding of the complexity and challenges of germline GS in trio contexts will also inform inclusion of trios in future precision medicine programs. Our primary aim was to explore the perspectives and experiences of healthcare professionals toward a trio germline GS study for newly diagnosed childhood cancer, including barriers to recruitment. A secondary aim was to assess healthcare professionals' genetics/genomics competency and educational needs.

## 2 Methods

We conducted this study in accordance with the Declaration of Helsinki (HREC approval: 2020/ETH00634).

### 2.1 Participants and Study Design

#### 2.1.1 PREDICT and PREDICT‐Impact

The Cancer PREDisposItion in Childhood by Trio‐based sequencing (PREDICT) Study is an Australian state‐based (NSW) multicenter germline GS study for cancer predisposition in sequentially newly diagnosed pediatric and adolescent patients with cancer [31]. Only P/LPVs in a curated gene list of cancer predisposition genes were reported, regardless of zygosity and inheritance [31]. PREDICT‐Impact is the prospective longitudinal mixed‐methods psychosocial component of PREDICT. In PREDICT‐Impact, we invited PREDICT pediatric oncology professionals (consultants and trainees) and genetic professionals (clinical geneticists, genetic counselors, and genetic nurses) to participate in an annual online questionnaire.

### 2.2 Procedure

PREDICT ran across three NSW‐based tertiary hospitals. The Sydney Children's Hospital and Children's Hospital at Westmead sites received governance approval in March 2021, followed by John Hunter Hospital in May 2022. Healthcare professionals delivering PREDICT were invited to participate via a personalized email invitation from the PREDICT‐Impact study coordinator (JDH). Participants opted out by reply email. Nonrespondents were sent reminders at 2‐ and 6‐week intervals Those who did not respond after three failed attempts were deemed unreachable but eligible the following year if they were still involved with delivering PREDICT.

Each recruiting site maintained screening logs that documented reasons eligible families considered for recruitment were not enrolled on PREDICT. A healthcare professional at each site involved with recruitment maintained this log, obtaining data from treating clinicians, families, and study records, where possible. All patients newly diagnosed with cancer were eligible for PREDICT, but not all eligible patients were considered for recruitment. Patients with pediatric cancers not requiring chemotherapy were not considered as they rarely attended pediatric oncology clinics, limiting opportunities to enroll them on PREDICT. Pathways for referral onto the PREDICT study also differed slightly across sites based on differing clinical processes.

### 2.3 Questionnaire Measures

The questionnaire was developed and pilot tested with a multidisciplinary expert panel. It included purposefully developed items based on available literature and expert opinion, adapted to the study context, and open‐ended qualitative questions. Questions explored perspectives and experiences of PREDICT, including perceived parent motivations [32, 33, 34], benefits, concerns [32, 34], and barriers, and assessed genetics/genomics competency and training needs: knowledge [16, 35, 36], confidence [16], education, and training preferences (Table 1). Only healthcare professionals who self‐identified as working in a clinical role on PREDICT completed perspectives and experiences of items.

**TABLE 1:** Questionnaire measures.

Perspectives and experiences of PREDICT		
Perception of parents' motivations for participating	6 items For example, “parents participate because they hope it will provide peace of mind”	5‐point Likert scale, 1 = not at all true, 5 = extremely true
Perception of study‐related concerns	5 items e.g., “I am concerned results will take a long time to come back”	5‐point Likert scale, 1 = not at all true, 5 = extremely true
Qualitative prompts	Perceptions of potential benefits of the study, study‐related concerns, and experienced barriers to recruitment—3 items For example, “Have you experienced any barriers to involving families in the PREDICT study?”	Open ended

Genetics/genomics competency and educational needs		
Knowledge	7 items For example, “My knowledge about general genetics is …”	4‐point Likert scale, 1 = very good, 4 = very poor
Confidence	5 items For example, “ability to explain germline genetic concepts to patients”	4‐point Likert scale, 1 = not confident at all, 4 = very confident, N/A = not part of my role
Personal education and training needs	List of topics related to genetic/genomic testing in childhood cancer care For example, “cancer genomics”	Select all that apply
Trainee education and training needs	List of topics related to genetic/genomic testing in childhood cancer care For example, “cancer genomics”	Select all that apply
Personal training preferences	List of possible options for delivering education/training for myself For example, “online webinars and courses”	Select all that apply
Trainee training preferences	List of possible options for delivering education/training to trainees in my profession For example, “online webinars and courses”	Select all that apply

### 2.4 Data Analysis

We present a cross‐sectional analysis of each unique participant's first response to the annual questionnaire, with qualitative open‐ended response data provided by unique participants at any questionnaire time point. We chose not to analyze data longitudinally, given the movement of some health professionals (e.g., fellows) between sites and the step‐wise opening of PREDICT across sites.

A quantitative‐dominant mixed‐methods approach was utilized with qualitative data examined to enhance understanding of quantitative results [37]. Quantitative and qualitative data were then compared side by side and integrated in the results [38].

Quantitative data was analyzed using descriptive statistics with the Statistical Package for the Social Sciences (version 28.0.1.0) [39]. Where answers were missing due to incomplete questionnaires or skipping of individual questions, denominators included respondents only. To assess differences between professions for categorical variables for knowledge, confidence, education, and training, we used Wilcoxon's rank‐sum (Mann–Whitney *U*) test and Fisher's exact test with a two‐tailed *p*‐value.

Qualitative data were analyzed using inductive content analysis [40]. One author (J.D.H.) read and familiarized herself with the data, followed by a first round of coding in Excel using an inductive approach to develop broad categories. These categories were refined in a second round of coding, and sub‐categories were developed. A second psychosocial researcher (K.H.) conducted a quality check on the coded content.

## 3 Results

### 3.1 Participants

Seventy healthcare professionals were invited to participate, of whom 33 opted in by completing at least one questionnaire (47% response rate), including 23 oncology professionals and 10 genetic professionals, mostly female (73%) (Table 2). Of these 33 unique participants, 23 self‐identified as working in a clinical role on PREDICT (23/33, 70%), with the remainder in research‐based or supportive roles.

**TABLE 2:** Demographics of participating healthcare professionals.

	*N* = 33
Gender, no. (%)	
Female	24 (73)
Age, years, no. (%)	
25–34	11 (33)
35–44	10 (30)
45–54	6 (18)
55+	6 (18)
Profession, no. (%)	
Oncology professionals	23 (61)
Genetic professionals	10 (26)
Self‐identified as working in a clinical role, no. (%)	
Yes	23 (70)
No	10 (30)
Time spent working in profession, years	
Mean (SD)	13 (11)
Range	> 1–42
Time spent working in pediatric oncology, years	
Mean (SD)	9.9 (10)
Range	> 1–38
Self‐reported formal genetics/genomics training, no. (%)	
Yes	14 (42)
No	19 (58)

Abbreviation: SD, standard deviation.

### 3.2 Healthcare Professionals' Perspectives and Experiences of PREDICT

#### 3.2.1 Parent Motivations and Benefits to Participation

**FIGURE 1:**
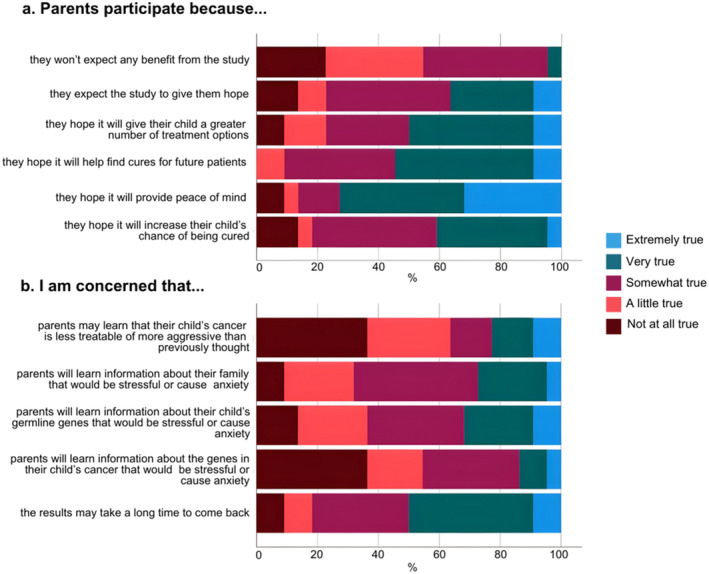
Healthcare professionals' (a) perceptions of parents' motivations for participation in PREDICT (b) and perceived concerns about PREDICT (*n* = 22).

Healthcare professionals' perceived parents as motivated to participate in PREDICT to gain benefits for both themselves and future families, with most agreeing parents participated to help find cures for future patients (*n* = 22/22, 100%), provide peace of mind (*n* = 20/22, 91%), increase their child's chance of cure (*n* = 19/22, 86%), give their child more treatment options (*n* = 20/22, 91%), and give them hope (*n* = 19/22, 86%) (Figure 1).

Qualitative data (Table 3) highlighted healthcare professionals' perception of the multiple potential benefits of PREDICT for future and participating families. They described benefits for future families by increasing knowledge and scientific evidence, improving equity of access to testing, and improving diagnostic yields of germline P/LPV in cancer predisposition genes:

When applied to all children with cancer we capture germline findings missed due to clinical panels targeted to known phenotypes. (Genetic counsellor, ID37)

**TABLE 3:** Categories and representative quotes regarding healthcare professionals' perspectives and experiences of PREDICT.

Benefits		
Categories and subcategories		Representative quotes
Benefits to future families and researchers	Knowledge gain	“Building knowledge about genetics, cancer predisposition syndromes, germline samples, inheritance among patient and clinical groups” (genetic counselor, ID37)
	Improve diagnostic yield	“We know that all children (patients) with underlying CPS will not be picked by testing which is done based on clinical criteria … we may pick up patients without significant clinical findings who have CPS … This may help in their clinical management” (pediatric oncologist, ID1)
	Equity	“There are many advantages. I would say the main one is giving the family the opportunity for genetic testing that they otherwise wouldn't have access to or would have to pay for” (genetic counselor, ID53) “Equity of access to genetic testing” (oncology fellow, ID12)
Benefits to family	Clinical	“Possible targeted treatment, risk management/early diagnosis of other cancer types for the patient, parents and relatives” (genetic counselor, ID37) “Improve early detection of subsequent cancers. Potential better management” (clinical geneticist, ID38)
	Psychological	“It can give the family a sense of control and choice over an aspect of their child's care and can give information about their own cancer risk as well as other family members” (genetic counselor, ID53)

Concerns		
Categories and subcategories		Representative quotes
Psychological challenges for families	Result implications	“Identifying germline mutations that can cause feelings of guilt, anxiety and grief” (oncology fellow, ID62) “The difference between ‘inconclusive’ and ‘negative’ needs to be clearly explained” (genetic counselor ID53) “Potential to find an abnormality with unclear significance” (pediatric oncologist, ID21)
	Study timing/design	“For some families, the time of diagnosis (or the two months following) is not an optimal time for them to be considering the chance that they, or their child (or other children) may be at a higher risk of cancer” (genetic counselor, ID37) “Germline results often have broader ramifications than just the proband/patient at hand. This is particularly true of a trio sequencing setup, which is designed to potentially diagnose parents even when they may not be anticipating their own cancer predisposition syndrome” (clinical geneticist, ID13)
Workforce	Logistics	“Large volume of information for informed consent, difficulties in collecting family history and samples, additional clinical load on staff already stretched” (oncology fellow, ID8)
	Clinician confidence	“Mainly being delivered by an oncology work force that is variably familiar, interested and comfortable with genomic testing” (clinical geneticist, ID13)
Ethical and privacy implications		“Ethical issues/interventions do not keep up with knowledge” (pediatric oncologist, ID3) “Insurance” (pediatric oncologist, ID20)

Barriers		
Categories and subcategories		Representative quotes
Procedural	Timing	“Timing has been the main barrier. Capturing the families far enough away from diagnosis, but within the time period is tricky. Also, working out when patients are in clinic/admitted and it is an appropriate time to approach” (pediatric oncologist, ID20)
	Logistics	“Language barriers and difficulty obtaining informed consent even with use of translator (plus additional difficulties accessing face to face or phone translators during COVID lockdown)” (oncology fellow, ID9) “Practical issues such as accessing rural patients who are not visiting the center frequently i.e. returning consent forms” (genetic counselor, ID70)
Clinician factors	Reluctance	“In my experience families are open to the idea and are interested to participate. There may be some reluctance on clinician's side to enroll patients” (pediatric oncologist, ID1).
	Limited genetic experience	“Given the mainstreaming model, sometimes there's challenges with non‐genetics clinicians recruiting the patients” (genetic counselor, ID36)
Family factors	Overwhelmed or uninterested	**“**Consent burden (newly diagnosed families are offered participation in many clinical trials)” (pediatric oncologist, ID1)
	Concern regarding result implications	“Concern re: germline analysis and insurance implications” (pediatric oncologist, ID26)
No barriers experienced		“No, fortunately, all families I have enrolled have been keen to participate” (pediatric oncologist, ID47)

Abbreviation: CPS, cancer predisposition syndrome.

Benefits for participating families were qualitatively described as clinical and psychological. Healthcare professionals described testing as potentially able to guide cancer treatment or surveillance in the child or their wider family and provide relief from anxiety or a sense of control:

Having given back ‘Normal or NAD’ results to a very anxious family—it was an amazing amount of information to be able to supply to them. (Pediatric oncologist, ID65)

#### 3.2.2 Concerns

Most healthcare professionals were concerned that participation in PREDICT would cause parents to learn information about their family (*n* = 20/22, 91%) or their child's germline genes (*n* = 19/22, 86%) that would be stressful or cause anxiety and that results could take a long time (*n* = 20/22, 91%) (Figure 1). Fewer were concerned that parents would learn information about the genes in their child's cancer that would be stressful or cause anxiety (*n* = 14/22, 64%) or that their child's cancer is less treatable or more aggressive than previously thought (*n* = 14/22, 64%).

Healthcare professionals qualitatively elaborated on how PREDICT may cause stress or anxiety for families:

Possibility of learning about inherited germline mutations which can impart a sense of guilt for parents, or stress/anxiety about possible other malignancies, or malignancies in other family members. (Oncology fellow, ID 62)

It was not just positive germline cancer predisposition findings healthcare professionals perceived as challenging. They also highlighted concerns regarding uncertain, secondary, or negative germline results:

I think a disadvantage is families may be too reassured if no variants are found… (Genetic counsellor, ID53)

Concerns regarding the PREDICT study design were also highlighted in qualitative data. Family distress could be compounded when testing is initiated at diagnosis for those who may be “overwhelmed with information” (*oncology fellow, ID9*) during this time, and when offered as trio testing:

By its very nature, it is a difficult to create truly informed consent for trio testing of such a broad panel, but even more so in the acutely heightened trauma setting of a childhood cancer diagnosis. (Clinical geneticist, ID13)

Concerns about workforce capacity were also highlighted. These included logistical challenges and variable clinician familiarity and confidence with germline testing, particularly regarding the return of results:

One of the biggest challenges has been the delivery of results—oncologists are probably not used to this being incorporated into their clinical care … Perhaps there is an under appreciation of the significance of a ‘negative’ result for families, and so disclosure of these types of results is not prioritized. (Genetic counsellor, ID36)

#### 3.2.3 Barriers to Recruitment

**FIGURE 2:**
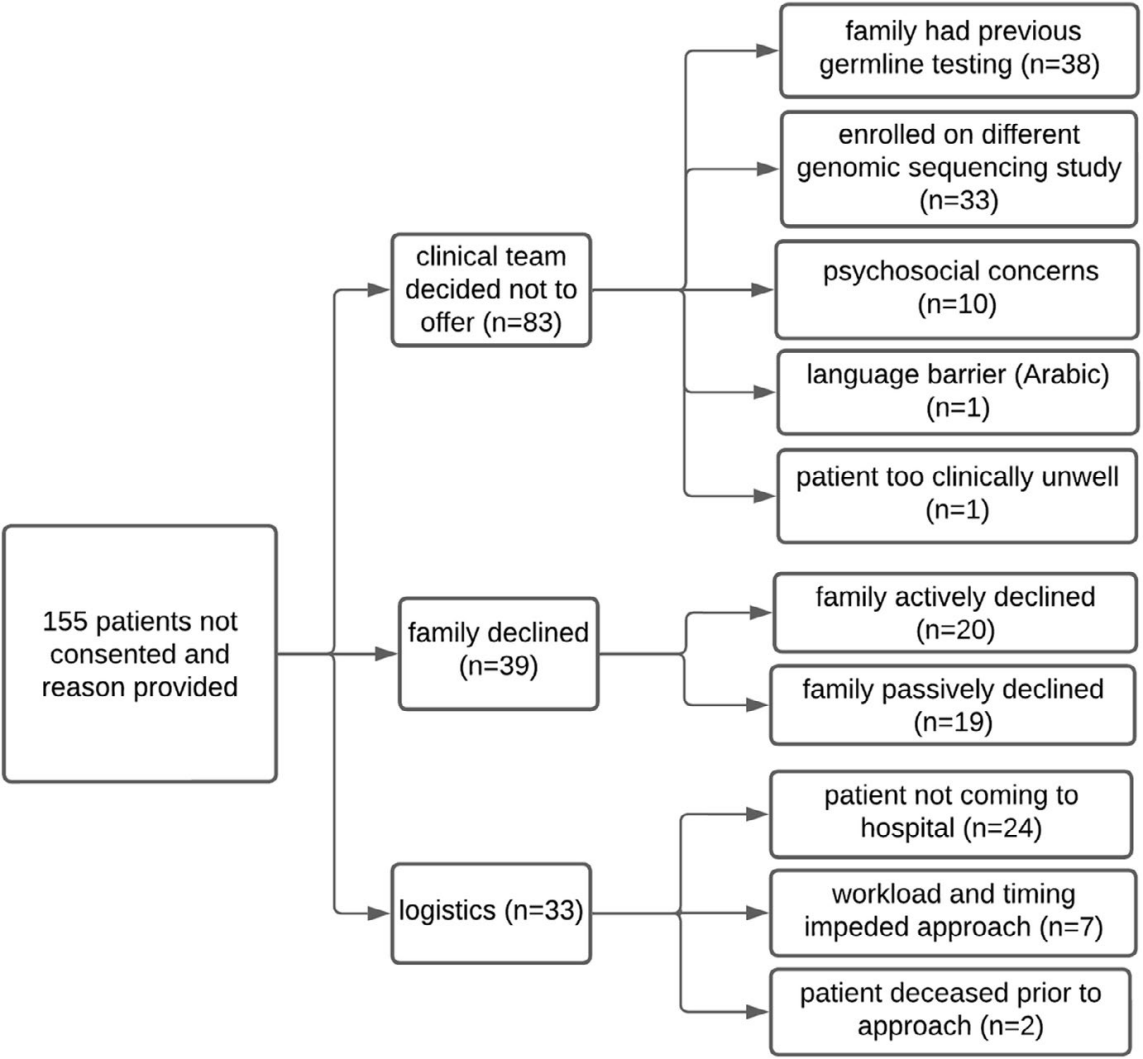
Recorded reasons eligible patients considered for recruitment were not enrolled in PREDICT as per study site records.

Screening logs at each recruiting site were used to record reasons why 155 eligible families were not enrolled in PREDICT. Reasons fell into three broader categories: clinical team decided not to offer (*n* = 83/155, 54%), family declined (*n* = 39/155, 25%), and logistics (*n* = 33/155, 21%) (Figure 2). On the basis of these records, clinical teams did not offer testing primarily because the family had already previous germline testing (*n* = 38) or were enrolled in a different GS study (*n* = 33). Ten families were not offered enrollment due to psychosocial concerns, one due to a language barrier, and another due to the patients' clinical status. Qualitative data collected through questionnaires highlighted how oncology professionals' limited experience with genetics may contribute to recruitment challenges or reluctance to enroll families related to psychosocial concerns:

Protectiveness on behalf of the treating clinician not wanting to overwhelm parents at an already stressful time. (Genetic counsellor, ID70)

Families who declined participation according to site records did so actively (e.g., indicated they did not want to participate, *n* = 20) or passively (e.g., did not return consent form, *n* = 19). Healthcare professionals' qualitatively described reasons for family refusal as related to feeling overwhelmed or uninterested at recruitment, or “parental concerns about impacts of positive findings, if identified” (oncology fellow, ID 9), including life insurance implications.

Logistical reasons for not enrolling eligible families according to site records included the patient no longer attending the recruiting hospital (*n* = 24), clinician workload, and timing impeded the approach to enrollment (*n* = 7), and for two families, the patient died before recruitment. Qualitative data collected in questionnaires closely reflected these logistical issues. Healthcare professionals described timing challenges as they struggled to find time to go through lengthy consents or find the right time to discuss enrollment with families who recently received a diagnosis. Logistical difficulties associated with consent and sample collection were also emphasized and described as exacerbated by additional external factors, including the use of interpreters, rurality, parent relationship status, and the COVID‐19 pandemic:

Difficulties obtaining parental bloods, especially in COVID lockdown and limitations on hospital visitors or when parents separated. (Oncology fellow, ID9)

### 3.3 Healthcare Professionals' Genetics/Genomics Competency and Educational Needs

#### 3.3.1 Knowledge and Confidence

**FIGURE 3:**
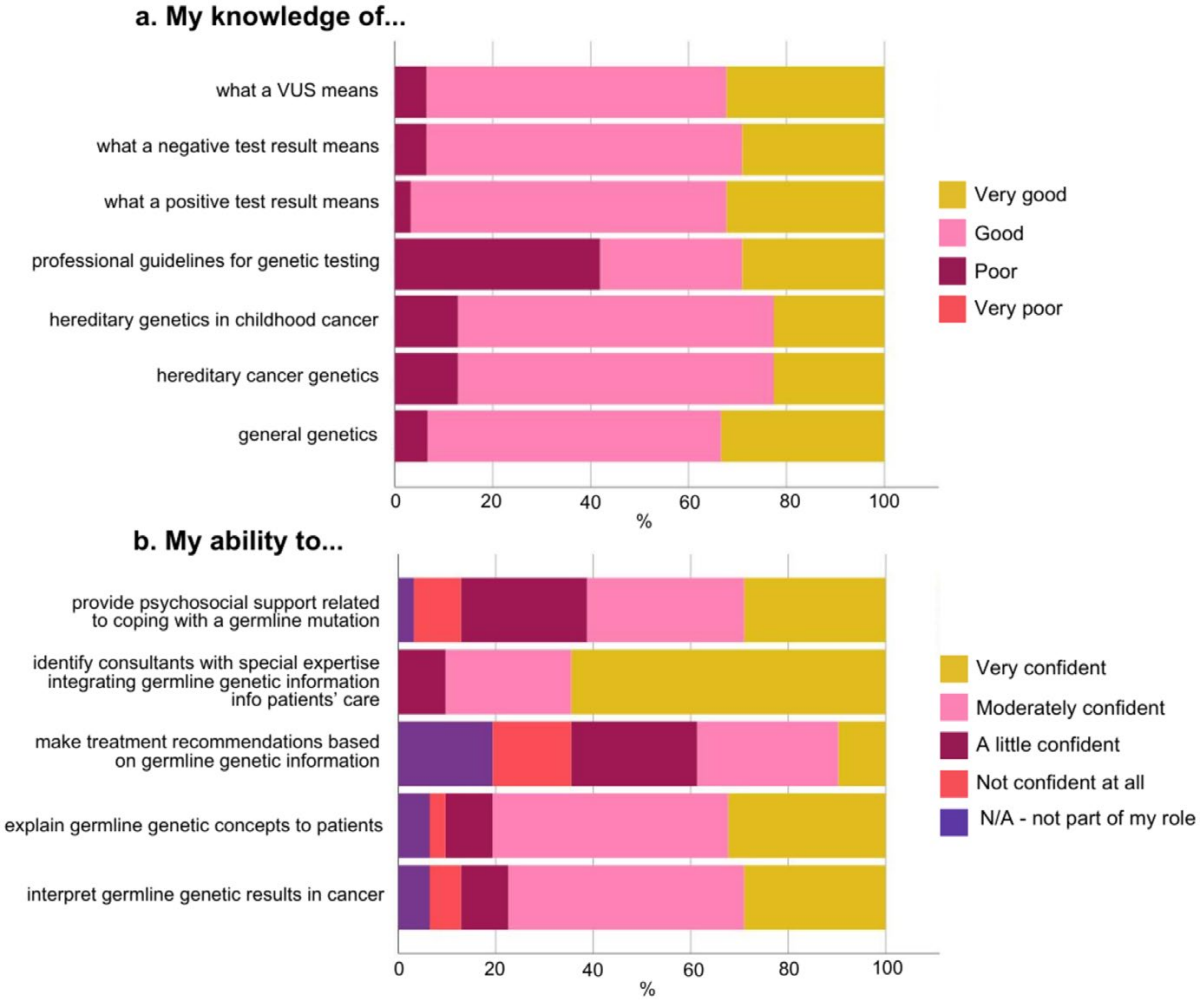
Healthcare professionals' self‐rated (a) knowledge and (b) confidence with genetics and genomics (*n* = 30 for “my knowledge of general genetics,” *n* = 31 for all other items). VUS, variant of uncertain significance.

No healthcare professionals rated their knowledge as “very poor” in any knowledge domain (Figure 3). Most indicated their knowledge was at least “good” in general genetics (*n* = 28/30, 93%), hereditary cancer genetics (*n* = 27/31, 87%), hereditary genetics in childhood cancer (*n* = 27/31, 87%), the meaning of a positive result (*n* = 30/31, 97%), the meaning of a negative result (*n* = 29/31, 94%), or a variant of uncertain significance (*n* = 29/31, 96%). Fewer reported their knowledge of professional guidelines for genetic testing as “good” or “very good” (*n* = 18/31, 58%), with 42% (*n* = 13/31) rating this domain “poor.” Genetic professionals rated themselves as more knowledgeable than oncology professionals across all domains (*p* = < 0.05 for all, Table S1).

Most healthcare professionals were “very confident” in their ability to identify consultants with special expertise in integrating germline genetic information into patient care (*n* = 20/31, 65%) (Figure 3). A minority were “very confident” in the following domains related to germline results: interpreting (*n* = 9/31, 29%), explaining to patients (*n* = 10/31, 32%), making treatment recommendations (*n* = 3/31, 9.7%), and providing psychosocial support (*n* = 9/31, 29%). Genetic professionals rated themselves as more confident than oncology professionals in their ability to interpret (*p* = 0.005), explain (*p* = 0.04), and provide psychosocial support related to germline results (*p* = < 0.001) (Table S1).

#### 3.3.2 Education and Training

Most healthcare professionals endorsed a need for education/training for trainees in their profession in all presented areas (Table S2). Fewer indicated that they would like education/training for themselves. Only 11/31 (36%) wanted education/training in the psychosocial implications of genetic/genomic testing or providing psychosocial support to families with a cancer predisposition. Most oncology professionals were interested in receiving education/training in understanding and interpreting germline results (13/22, 59%), ethical implications (13/22, 59%), and legal implications of testing (12/22, 54%), whereas most genetic professionals wanted training in understanding and interpreting somatic results (5/9, 56%). We checked for significant associations between profession and item responses and found none (Table S1).

When asked how they would like training for themselves delivered, most endorsed professional development workshops (21/31, 68%), departmental presentations (17/31, 55%), or external seminars/conferences (17/31, 55%). A minority endorsed training for themselves via specialty written resources (13/31, 42%) and participation in multidisciplinary tumor boards (14/31, 45%), online webinars/courses (15/31, 48%), or formal certification/fellowship activities (6/31, 19%). For trainees in their profession, most endorsed delivery via departmental presentations (27/31, 87%) or professional development workshops (27/31, 87%) and participation in multidisciplinary tumor boards (25/31, 81%), external conferences and seminars (25/31, 81%), specialty resources (22/31, 71%), and online webinars/courses (23/31, 74%). Fewer believed training should be via formal certification (14/31, 45%).

## 4 Discussion

Our study offers valuable insights into current obstacles to implementing routine germline GS into childhood cancer care, with unique insights for delivering standalone trio testing at diagnosis. Reflecting findings in precision medicine research [14, 16], healthcare professionals expressed positive attitudes toward testing and perceived parents as motivated to participate for potential benefits for their child and future families, aligning with previous parent‐reported motivations [5, 32, 34, 41]. Benefits were described as clinical and psychological, including relief and peace of mind. Some health professionals emphasized the relief a “no findings” result could provide families, whereas others cautioned it might mistakenly suggest no genetic link to the child's cancer. Although excluding known genetic causes may ease parental anxiety, overemphasis on these results could indicate misunderstanding of testing limitations among healthcare professionals. It is crucial that healthcare professionals and parents understand that “no findings” results may evolve with new evidence.

Despite these benefits, as in previous studies [13, 15, 16], healthcare professionals' worried participation would cause stress and anxiety in families. In some cases, psychosocial concerns resulted in the clinical team declining to offer enrolment. These concerns centered around implications of trio testing, which may unexpectedly diagnose parents primarily focused on their child's treatment, and the timing of testing at cancer diagnosis. Parents may decline participation in germline testing for their child with cancer out of fear or guilt related to uncovering their own genetic status or feeling overwhelmed at diagnosis [42]. These concerns could be mitigated by conducting initial singleton germline testing on the child after allowing time to adjust to the diagnosis, particularly when testing is not clinically urgent [43]. Although hindering rapid inheritance assessments, this may increase family uptake and ease clinician concerns, with parental testing pursued if a reportable P/LPV finding is identified.

Alternatively, targeted training for clinicians in delivering trio testing could enhance their comfort and confidence, along with ongoing research into the psychological impacts on families. This would enable the development of resources and interventions to ensure that serious negative psychological outcomes remain infrequent [42, 44, 45].

Additional barriers to recruitment in our study related to logistical challenges and clinician factors. Healthcare professionals described difficulties collecting samples, obtaining consent, and gathering family history, finding time and space to have lengthy consent discussions, and that discussions are often performed by oncology professionals with variable levels of experience with germline GS. Logistical challenges were likely exacerbated by the trio context, requiring the collection of both parent and child consents and samples, providing further justification against an upfront trio approach. Additionally, previous studies have identified logistics, resource limitations, and low oncology clinician confidence as barriers to integrating genetics and genomics into clinical practice [46]. Resources like online consent modules and educational materials for families could help address these challenges, easing the consent burden and reducing the workload for clinicians.

Most healthcare professionals rated their genetics knowledge as “good,” but few felt “very confident” in performing tasks related to delivering germline GS to families, consistent with previous literature [16, 22]. Unsurprisingly, given their training, genetic professionals rated themselves higher than oncology professionals in all knowledge domains and confidence in providing psychosocial support, interpreting germline results, and explaining germline genetic concepts to patients. However, oncology professionals are required to perform consent and return of result discussions with families, necessitating the development of these skills. Oncology professionals in our study, like those in previous literature [25, 26], may instead be continuing to rely on genetic professionals to support these processes. Genetic professionals delivering precision medicine for children with high‐risk cancer have identified a high demand for their skills [14], which is likely to increase [28]. It is unsustainable to expect genetic professionals to continue to meet these ever‐increasing demands. Additionally, although most oncology and genetic professionals recognized a need for dedicated genetics/genomics education/training for trainees, fewer were interested in further education/training for themselves. Low confidence in, or desire for, training in psychosocial domains related to germline GS could indicate a gap in current models of care, which may need to be filled by other allied health professions.

### 4.1 Limitations and Future Directions

The small sample size, resulting from a low response rate, restricted analyses to descriptive and exploratory statistics and limited generalizability of findings. However, our response rate is comparable to other healthcare professional questionnaire studies, which typically report rates between 40% and 50% [47, 48]. The sample included a high proportion of women, likely skewed by the inclusion of genetic professionals who in Australia are mostly women [28, 49]. Additionally, the varying participant group and step‐wise opening of PREDICT limited our ability to assess changes over time. Knowledge and confidence may improve with increased experience delivering germline GS, and further studies should explore impacts of “on the job” or experiential learning [50]. Our sample may also include selection bias, attracting respondents supportive of germline GS, resulting in more positive attitudes. These respondents may be relative experts in genetics/genomics, biasing data on knowledge/confidence and education/training needs. Further studies should explore attitudes of those who may not support germline GS in childhood cancer care to better understand their reservations and needs.

## 5 Conclusion

Healthcare professionals delivering germline GS for children with cancer recognize testing holds potential clinical and psychological benefits for participating and future families, which drives parent participation. However, challenges to successful uptake in routine practice persist. Careful consideration of recruitment timing is crucial to avoid overwhelming families who may already be distressed by their child's cancer diagnosis. Alternatives to trio testing may reduce the complexity of informed consent discussions and mitigate parental and clinician concerns regarding potential psychological impacts of uncovering parental genetic status. Addressing logistical and resourcing barriers is essential and will require targeted strategies, such as online consent options, to alleviate system and personnel pressures. Genetic professionals face ever‐increasing and unsustainable demand for their expertise. Genetic professionals could be supported in their delivery of psychosocial care by other allied health professionals, including nurses, psychologists, and social workers, to help alleviate these pressures. Models of care delivering testing should consider clearly outlining and coordinating the roles and responsibilities of all professionals involved.

## Author Contributions

**Jacqueline D. Hunter:** conceptualization (lead), data curation (lead), formal analysis (lead), funding acquisition (equal), investigation (lead), methodology (lead), project administration (lead), visualization (lead), writing – original draft (lead), writing – review and editing (lead). **Kate Hetherington:** conceptualization (supporting), funding acquisition (equal), methodology (supporting), project administration (supporting), supervision (equal), writing – review and editing (supporting). **Claire E. Wakefield:** conceptualization (supporting), funding acquisition (equal), methodology (supporting), writing – review and editing (supporting). **Katherine M. Tucker:** conceptualization (supporting), funding acquisition (equal), methodology (supporting), writing – review and editing (supporting). **Brittany C. McGill:** conceptualization (supporting), funding acquisition (equal), methodology (supporting), writing – review and editing (supporting). **Andrew Grant:** data curation (supporting), writing – review and editing (supporting). **Noemi A. Fuentes‐Bolanos:** conceptualization (supporting), data curation (supporting), funding acquisition (equal), supervision (equal), writing – review and editing (supporting). **Bhavna Padhye:** data curation (supporting), funding acquisition (equal), writing – review and editing (supporting). **Margaret Gleeson:** data curation (supporting), writing – review and editing (supporting). **Kanika Bhatia:** funding acquisition (equal), supervision (equal), writing – review and editing (supporting). **Michelle Peate:** conceptualization (supporting), funding acquisition (equal), methodology (supporting), supervision (equal), writing – review and editing (supporting).

## Conflicts of Interest

K.M.T. has received an honorarium for preparing educational material on Von Hippel–Lindau disease and for participating in a committee to progress access to medical treatments for Von Hippel–Lindau disease in Australia. All other authors have declared no conflicts of interest.

## Supporting information


Data S1.

## Data Availability

The data that support the findings of this study are available on request from the corresponding author. The data are not publicly available due to privacy or ethical restrictions.
