# Supplementary material for: Delivering Trio Germline Whole Genome Sequencing to Patients Newly Diagnosed With Childhood Cancer: Healthcare Professionals' Perspectives of the PREDICT Study
Source: Cancer Med. 2025 Feb 14;14(4):e70680. doi: 10.1002/cam4.70680 (PMC11829071; doi:10.1002/cam4.70680)
Supplement: Supplementary file 1 — Data S1. [file CAM4-14-e70680-s001.docx]

**Supplementary Materials**

**Table S1.** **Comparison of knowledge and confidence ratings by profession using Wilcoxon’s rank-sum (Mann-Whitney U) tests.**

|  | Oncology professionals: median (min-max) | Genetics professionals: median (min-max) | p-value |
| --- | --- | --- | --- |
| **My knowledge of….** |  |  |  |
| genetics in general | 3 (2-4) | 4 (3-4) | .01 |
| hereditary cancer genetics | 3 (2-4) | 4 (3-4) | .002 |
| hereditary genetics in childhood cancer | 3 (2-4) | 4 (3-4) | .002 |
| professional guidelines for genetic testing | 2 (2-4) | 4 (2-4) | .001 |
| what a positive test result means | 3 (2-4) | 3 (3-4) | .001 |
| what a negative test results means | 3 (2-4) | 4 (3-4) | <.001 |
| what a variant of uncertain significance means | 3 (2-4) | 4 (4-4) | <.001 |
| **My ability to…** |  |  |  |
| interpret germline genetic results in cancer | 3, (1-4) | 4 (3-4) | .005 |
| explain germline genetic concepts to patients | 3 (1-4) | 4 (3-4) | .04 |
| make treatment recommendations based on germline genetic information | 2.5 (1-4) | 2 (1-3) | .7 |
| identify consultants who have special expertise in integrating germline genetic information into patients care | 4 (2-4) | 4 (3-4) | .1 |
| provide psychosocial support related to coping with a germline mutation | 2 (1-4) | 4 (3-4) | <.001 |
| Note: Knowledge response options: 1 = very good, 4 = very poor, confidence response options: 1 = not confident at all, 4 = very confident. | | | |

**Table S2. Healthcare professionals’ genetic/genomic education and training needs.**

| **no. (%)** | **Training for trainees in my profession** | | | | **Training for myself** | | | |
| --- | --- | --- | --- | --- | --- | --- | --- | --- |
|  | Total N=31 | Oncology professionals (n=22) | Genetics professionals (n=9) | Fisher’s exact p-value (2-sided) | Total (N=31) | Oncology professionals (n=22) | Genetics professionals (n=9) | Fisher’s exact p-value (2-sided) |
| Cancer genomics | 30 (97) | 21 (96) | 9 (100) | 1.0 | 15 (48) | 11 (50) | 4 (44) | 1.0 |
| Cancer predisposition in childhood cancer | 30 (97) | 21 (96) | 9 (100) | 1.0 | 14 (45) | 10 (46) | 4 (44) | 1.0 |
| Understanding and interpreting germline results | 24 (77) | 16 (73) | 8 (89) | .6 | 17 (55) | 13 (59) | 4 (44) | .7 |
| Understanding and interpreting somatic results | 23 (74) | 15 (68) | 8 (89) | .4 | 16 (52) | 11 (50) | 5 (56) | 1.0 |
| Communicating results to patients and their families | 24 (77) | 17 (77) | 7 (78) | 1.0 | 12 (39) | 10 (46) | 2 (22) | .4 |
| Ethical implications | 27 (87) | 19 (86) | 8 (89) | 1.0 | 15 (48) | 13 (59) | 2 (22) | .1 |
| Legal implications | 25 (81) | 18 (82) | 7 (78) | 1.0 | 14 (45) | 12 (54) | 2 (22) | .1 |
| Psychosocial implications | 26 (84) | 18 (82) | 8 (89) | 1.0 | 11 (36) | 9 (41) | 2 (22) | .4 |
| Providing psychosocial support to families of children with an identified CPS | 24 (77) | 16 (73) | 8 (89) | .6 | 11 (36) | 8 (36) | 3 (33) | 1.0 |
| CPS = cancer predisposition syndrome | | | | | | | | |
